# COVID-19: the importance of physical therapy in the recovery of workers’ health

**DOI:** 10.47626/1679-4435-2021-709

**Published:** 2021-04-30

**Authors:** Luís Eduardo Santos Paz, Bruno José da Silva Bezerra, Taciane Machado de Melo Pereira, Welma Emidio da Silva

**Affiliations:** 1 Departamento de Fisioterapia, Faculdade de Integração do Sertão, Serra Talhada, PE, Brazil; 2 Departamento de Morfologia e Fisiologia Animal, Universidade Federal Rural de Pernambuco, Recife, PE, Brazil

**Keywords:** physical therapy, COVID-19, rehabilitation, workers’ health

## Abstract

Coronavirus disease 2019 (COVID-19) is likely to have a major impact on society and the economy since the illness is currently infecting a significant number of active workers in the industry and service sectors. The illness can have long-term consequences for patients, affecting their functional capacity and, consequently, their occupational performance. This study analyzed the effects of COVID-19 on occupational health, with a focus on the importance of physical therapy in rehabilitation. An integrative literature review was conducted based on articles retrieved from the PubMed, SciELO, and LILACS databases using the following keywords: COVID-19, physical therapy, rehabilitation, and occupational health. The search retrieved 1,308 studies, 15 of which met inclusion criteria for the review. A thorough assessment of the articles revealed four topics that corresponded to the results of this study: 1) effects of COVID-19 on occupational health; 2) physical therapy in mild and moderate cases without hospitalization; 3) physical therapy in hospitalized patients with COVID-19; 4) physical therapy in post-intensive care unit (ICU) recovery and after hospital discharge. The findings showed that COVID-19 can affect several physiological systems and have both short- and long-term effects on patients, including physical and psychological impairments. Physical therapists must be involved in the battle against this illness to help patients recover their physical function and return to work as quickly, safely, and effectively as possible.

## INTRODUCTION

Coronavirus disease 2019 (COVID-19) is an illness caused by infection with the novel coronavirus (severe acute respiratory syndrome coronavirus 2 [SARS-CoV-2]), which was first identified in a December 2019 outbreak in the city of Wuhan, China, before quickly moving on to several countries across the world.^1^ The rapid global spread of this illness, combined with its high infection and death rates, prompted the World Health Organization (WHO) to deem it a public health emergency of international concern on January 30, 2020, and declare a pandemic on March 11 of the same year.^2,3^

The SARS-CoV-2 is a single-stranded RNA virus in the *Coronavirida*e family. The pathogens in this family affect humans and a variety of other animals, causing infections that range from asymptomatic to very severe.^4^ In humans, the mild or oligosymptomatic form of this virus infection is associated with clinical manifestations such as fever, cough, rhinorrhea, sore throat, anosmia, ageusia, asthenia, mild dyspnea, hyporexia, nausea, diarrhea, and vomiting.^5^ However, the infection can also progress to *severe acute respiratory syndrome* (SARS), leading to severe dyspnea, pneumonia, or even death.^6^

The effects of the coronavirus responsible for COVID-19 can extend far beyond the respiratory system, affecting the cardiovascular, renal, gastrointestinal, endocrine, nervous, and musculoskeletal systems.^7,8^ Older age and comorbidities such as smoking, obesity, diabetes mellitus, hypertension, heart disease and previous respiratory illnesses also constitute risk factors for more severe disease.^9^

Furthermore, patients with SARS usually require prolonged hospitalization and mechanical ventilation, which can result in serious side effects and the development of post-intensive care syndrome.^10,11^ This condition is characterized by physical, cognitive, and psychiatric impairments that affect patients’ quality of life even after hospital discharge.^12^

The literature shows that patients who developed SARS in a previous outbreak of coronavirus continued to display reduced respiratory capacity and musculoskeletal limitations years after the end of the disease.^13,14^ Additionally, asymptomatic or even non-infected individuals can experience reduced functional capacity due to a sedentary lifestyle during social distancing; this is especially likely in individuals with pre-existing musculoskeletal diseases.^15^ These data support the conclusion that COVID-19 can have direct and indirect effects on health, both in the short- and the long term. COVID-19 can undoubtedly have a major impact on society and the economy since the novel coronavirus has infected a significant number of active workers in the industry and service sectors, especially those working in health care settings.^16^

Physical therapists play an important role in combating the COVID-19 pandemic, as they contribute to the prevention and rehabilitation of impairments caused by the illness,^15^ in addition to assisting with functional independence and facilitating individuals’ reintegration into society and the job market. This study aimed to analyze the effects of COVID-19 on occupational health, with a focus on the importance of physical therapy in rehabilitation.

## METHODS

The goals of this study were achieved through a literature review. The review involved the following steps: topic identification, definition of the research question, methodological design, definition of inclusion and exclusion criteria, data collection and selection, evaluation of included studies, interpretation of the results, and presentation of the review. Articles were identified in the United States National Library of Medicine (PubMed), Scientific Electronic Library Online (SciELO), and Latin American & Caribbean Health Sciences Literature (LILACS) databases in August 2020, using the following Health Sciences Descriptors (DeCS): COVID-19, physical therapy, rehabilitation, and occupational health, in both Portuguese and English. The terms were combined using the Boolean operator “AND” to locate studies that contained all relevant keywords.

**Figure 1:**
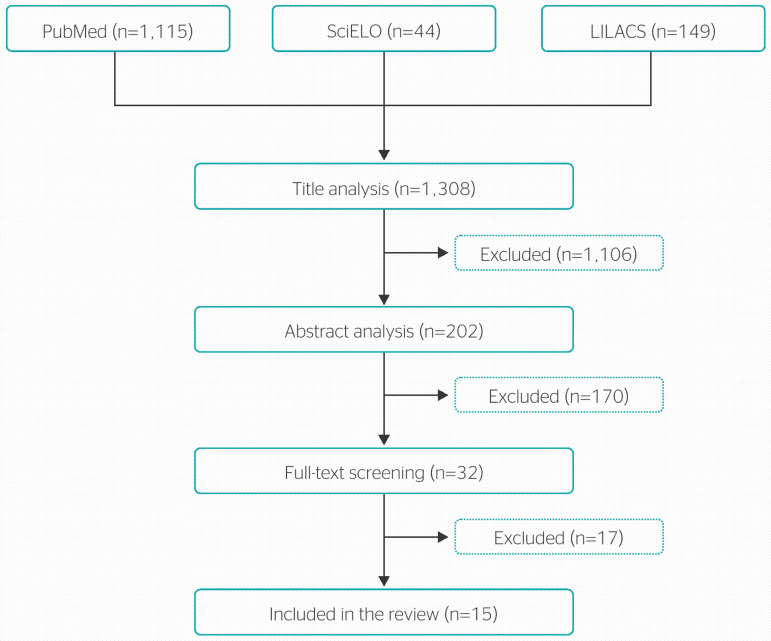
Literature search and study selection flow chart. LILACS = Latin American & Caribbean Health Sciences Literature; PubMed = United States National Library of Medicine; SciELO = Scientific Electronic Library Online.

The following inclusion criteria were set for the review: full-text availability; English, Spanish, and/or Portuguese language; having “consequences of COVID-19”, “COVID-19 and physical therapy,” and “COVID-19 and occupational health” as the main topic of study. Duplicate studies and those that did not address the theme of the study were excluded. The reference lists of the main articles included in the review were also hand-searched to identify any relevant studies missed by the electronic search. The search retrieved a total of 1,308 articles across all databases. After title analysis and the application of inclusion criteria, 202 studies were selected for abstract screening. Thirty-two studies were then selected for full-text reading, which led to the exclusion of 12 studies. As a result, the final sample consisted of 15 articles. Figure 1 shows the search and selection strategies used in this review.

## RESULTS AND DISCUSSION

The 15 articles in the review comprised 7 studies conducted in China, 3 in the United States, 2 in Brazil, 1 in Italy, 1 in England, and 1 multicenter study. Seven studies were descriptive, 2 were literature reviews, 2 were experience reports, 2 were official documents, 1 was a descriptive study and 1 was a quasi-experimental study (Table 1).

**Table 1:** Summary of articles reviewed, including authors, country, type of article, objectives, and relation to the theme of this review

Author	Country	Type of article	Objective	Relation to the theme
Wang et al.^17^	China	Descriptive and exploratory	To evaluate levels of psychological distress, anxiety, depression, and stress in the early stages of the COVID-19 outbreak.	COVID-19 and clinical consequences
Iannaccon et al.^18^	Italy	Experience report	To identify barriers to functional recovery in patients with COVID-19 and make recommendations for the development of standardized clinical procedures.	COVID-19 and rehabilitation
Zhou et al.^19^	China	Descriptive and exploratory	To analyze risk factors for hospital death and describe the clinical course of COVID-19 symptoms.	COVID-19 and clinical consequences
Rede CoVida^20^	Brazil	Literature review	To assess occupational health risks and vulnerabilities during the COVID-19 pandemic.	COVID-19 and occupational health
Guan et al.^21^	China	Descriptive and exploratory	To analyze the clinical characteristics of COVID-19.	COVID-19 and clinical consequences
Baker et al.^22^	United States	Descriptive and exploratory	To assess the number and occupational categories of workers exposed to infection and disease at their workplace.	COVID-19 and occupational health
Lan et al.^23^	United States	Descriptive and exploratory	To identify occupations with a high risk of local transmission of COVID-19.	COVID-19 and occupational health
Wu & McGoogan^24^	China	Descriptive and exploratory	To analyze all cases diagnosed before February 11, 2020.	COVID-19 and clinical consequences
Thomas et al.^25^	China	Recommendations	To standardize techniques and procedures for pulmonary rehabilitation.	COVID-19 and rehabilitation
PAHO^26^	United States	Official document	Considerations and recommendations for rehabilitation.	COVID-19 and rehabilitation
Halpin et al.^27^	England	Descriptive and exploratory	To examine the impact of COVID-19 on survivors after hospital discharge.	COVID-19 and clinical consequences
Liu et al.^28^	China	Quasi-experimental	To examine the effects of a 6-week pulmonary rehabilitation program on respiratory function, quality of life, mobility, and psychological functions in older adults with COVID-19.	COVID-19 and rehabilitation
Wang et al.^29^	China	Literature review	To serve as a guide and starting point for the management of functional impairments and comorbid issues associated with COVID-19.	COVID-19 and rehabilitation
Carda et al.^30^	Multicenter study	Experience report	To share the experiences and perspectives of clinicians in different rehabilitation centers for COVID-19.	COVID-19, clinical consequences and rehabilitation
Brasil^31^	Brazil	Epidemiological bulletin	Disclosure of epidemiological data and information on the structure established to combat COVID-19 in Brazil.	COVID-19, clinical consequences and occupational health

COVID-19 = coronavirus disease 2019; PAHO = Pan-American Health Organization.

The review showed that, despite the wealth of research on COVID-19 published in the past year, few studies have evaluated the effects of this illness on occupational health or the work of physical therapists in the rehabilitation of patients with COVID-19. However, some studies did provide clinical descriptions of patients with COVID-19. This was most frequent in studies performed in China, the epicenter of the disease. Experts, occupational associations, and professional organizations in several countries have also published recommendations for the treatment of these patients. These recommendations were based on the experience of countries that have faced or are facing a large number of COVID-19 cases; on previous research on the treatment of other coronavirus infections; and on studies of SARS not caused by a coronavirus.

After reading the articles, four main themes were identified and used to structure this review: 1) effects of COVID-19 on occupational health; 2) physical therapy in mild and moderate cases without hospitalization; 3) physical therapy in hospitalized patients with COVID-19; and 4) physical therapy in post-intensive care unit (ICU) recovery and after hospital discharge.

### EFFECTS OF COVID-19 ON OCCUPATIONAL HEALTH

The clinical presentation of coronavirus infections in humans can vary widely, ranging from a mild cold to severe illnesses such as those associated with the 2002 SARS-CoV epidemic and the Middle East respiratory syndrome coronavirus (MERS-CoV) outbreaks in 2012 and 2015.^32^ At the end of 2019, a new strain of coronavirus was found to be responsible for outbreaks of respiratory disease in humans. The pathogen was temporarily named the novel coronavirus 2019 (2019-nCoV). Genetic sequencing studies confirmed the existence of a new strain of coronavirus, which was classified as a member of the *Betacoronavirus* family and named SARS-CoV-2, marking the third instance in which a coronavirus jumps from animals to humans in less than two decades.^33,34^

Although human infection by SARS-CoV-2 is a recent phenomenon and its clinical features have not been fully defined, the virus is known to be highly pathogenic and cause both upper and lower respiratory tract infections with a risk of lethality.^4^ The infection is mediated by an affinity with the angiotensin-converting enzyme 2 (ACE2) receptor, which plays a fundamental role in the renin-angiotensin-aldosterone system and influences the regulation of arterial pressure and electrolyte homeostasis.^33,35^ ACE2 is highly expressed in lung cells and several extrapulmonary tissues, including gastrointestinal, cardiac, endothelial, skin, and smooth muscle tissue, as well as the oral and nasal mucosa.^36^ When infected, these tissues increase the production of ACE2 and immunosuppressant proteins, as well as the release of inflammatory cytokines.^34^ Since ACE2 is frequently expressed in type II alveolar cells, the lungs are often compromised in patients with COVID-19. However, the wide distribution of these receptors in other organs allows COVID-19 to affect several organ systems.^37^

Although patients can have multisystemic infections, the symptoms of COVID-19 can vary between patients, and in most cases, are classified as mild to moderate.^19^ Yet the infection can become severe, resulting in serious respiratory problems, viral pneumonia, and even death.^6^ The signs and symptoms of COVID-19 emerge on average 5 to 6 days after infection (mean period of incubation: 5-6 days, range: 1-14 days).^38^ The course of the illness is divided into two phases: the acute phase involves mostly respiratory symptoms, while the post-acute phase can include symptoms associated with prolonged immobilization, previous respiratory dysfunction, as well as cognitive and emotional disturbances.^18^

Studies show that older adults and obese individuals, as well as those with heart disease, immunosuppression, and preexisting respiratory problems, are more likely to develop severe forms of the disease.^9^ Recent studies have noted that, since SARS-CoV-2 binds to host cells through the peptidase domain of ACE2, which facilitates entry and replication,^33,35^ people treated with angiotensin-converting inhibitors and/or angiotensin II type I receptor blockers, such as patients with diabetes and hypertension, are also more likely to develop severe forms of COVID-19.^39^

In light of these observations, the impact of COVID-19 on human health is a cause for concern, since the illness affects a significant number of working-age and economically active individuals.^20^ A study conducted in China on 1,099 people with COVID-19 found that patients had a mean age of47 years.^21^ Similarly, a meta-analysis of clinical trials of COVID-19 involving 46,959 individuals infected by SARS-CoV-2 observed that patients had a mean age of 46.62 years and that 55.6% of patients were male (95% confidence interval).^40^.

The literature shows that infection rates were higher in certain occupational categories and that both occupational activities and working conditions could contribute to exposure to the virus and the spread of disease.^22,41^ At the start of the outbreak, employees and clients of a wet market in Wuhan, China, were among the first to be infected.^42^ In Singapore, 68% of the first locally transmitted cases were attributed to occupational activities.^41^ A study that evaluated confirmed cases of COVID-19 in six Asian territories (Hong Kong, Japan, Singapore, Taiwan, Thailand, and Vietnam) found that 14.9% of cases were associated with work and that the sectors most affected by COVID-19 were health care (22%), transportation (18%), services and sales (18%), cleaning and domestic work (9%) and public safety (7%).^(.4)^ These observations were corroborated by Lan et al.^23^ A report by the Centers for Disease Control and Prevention reported that 55% of hospitalizations and severe cases were reported in non-white people; the vulnerability of this population was attributed to inequalities in health and housing, as well as the nature of their working conditions.^43^

It is important to note that, in Brazil, underreporting affects both overall case numbers and estimates of the number of professionals on medical leave, infected or dead as a result of COVID-19. The first confirmed case of COVID-19 in Brazil was reported to the Ministry of Health (Ministério da Saúde; MS) on February 26 2020, but it was only on March 31 2020 that the item “occupation” was included in report forms for patients hospitalized with SARS, which are sent to the Influenza Epidemiological Surveillance System (Sistema de Informação de Vigilância da Gripe; SIVEP-Gripe).^20,31^ Nevertheless, data from the period of January to the first half of April 2020 revealed that occupation was only reported for 1.7% of the 53,733 cases notified to the SIVEP-Gripe.^20^ This interferes with occupational health surveillance and risk analysis in different professional categories, hindering the development of strategies to combat the illness in work environments. To date, few studies on COVID-19 have addressed working conditions and organization, with most investigations focusing on individual hygiene and social distancing protocols.^20,44^

Despite underreporting, the 36th Epidemiological Bulletin issued by the Brazilian MS alerted to the large number of SARS cases and deaths among health care workers. According to this document, by October 17 (at which point Brazil placed third in a global ranking of confirmed cases and second in the number of deaths), health care workers accounted for 23.6% (369,260) of reported cases of COVID-19. Although this does not represent the total number of health professionals infected in the country, it reflects the more severe cases in this population.^31^ Epidemiological reports from other countries also report the number of cases among health care workers as a percentage of total cases.^2,24,45^.

These data alert to the importance of occupational health, since studies of patients with SARS caused by previous strains of coronavirus or other viruses altogether have shown that patients can continue to experience physical symptoms (reduced cardiorespiratory function, musculoskeletal limitations, and psychological problems) long after the infection subsides, showing that the illness can have short-, medium- and long-term effects on patients.^13,14^ It is therefore crucial to develop and implement strategies to support workers in the recovery process since the available data already demonstrates a growing demand for specialized rehabilitation services by patients who recover from COVID-19.^15,18^

### PHYSICAL THERAPY IN MILD AND MODERATE CASES WITHOUT HOSPITALIZATION

The severity of COVID-19 can be classified into four levels: mild, moderate, severe and critical.^46^ According to the MS, most patients (80%) infected with SARS-CoV-2 are categorized as mild to moderate and have symptoms such as discomfort, fever, fatigue, coughing, mild dyspnea, anorexia, sore throat, generalized pain, headaches, nasal congestion, diarrhea, nausea, and vomiting.^5,16^ Hyposmia/anosmia and ageusia have also been reported.^47^

In mild cases, patients have influenza-like symptoms and normal radiological findings, which differs from moderate severity cases.^46^ It is important to note that fever may not always be present, especially in young or older patients, immunosuppressed individuals, or patients taking antipyretic medication.^48^ The literature shows that these patients can be treated by symptom management,^46^ and do not usually require hospitalization. However, they must be isolated at home for 14 days after symptom onset, taking care of their health and preventing the spread of disease. In this situation, non-pharmacological strategies can be used to prevent an aggravated clinical course.^48,49^

Rehabilitation professionals play a crucial role in the self-isolation period, helping patients optimize their functional independence and improve quality of life. Studies show that, during isolation, patients naturally spend more time sitting or lying down, which may contribute to exercise intolerance, reduced muscle strength, musculoskeletal symptoms such as myofascial pain and arthralgia, and an increased risk of deep vein thrombosis.^11^ The physical therapy protocol for these patients should include low-intensity aerobic exercises, muscle strengthening, balance training, as well as stretching.^50^ These recommendations are based on scientific evidence of the role of physical exercise in the strengthening of the cardiovascular and immune systems, as well as the physiological functions of the body.^51^

In patients with respiratory difficulties, physical therapists can help improve respiratory function and determine whether hospitalization is necessary based on the assessment of dyspnea and oxygen saturation through pulse oximetry (SpO_2_). According to the literature, respiratory exercises should be recommended in mild cases to assist with the improvement of respiratory health and disease prognosis.^52^ In patients with cough and difficulty expectorating sputum, secretion clearance techniques should be used. However, these are considered high-risk procedures, as they produce and spread microdroplets, which may increase the risk of transmission of SARS-CoV-2. Therefore, procedures and techniques that involve changes in respiratory flow and secretion drainage should only be considered after a careful risk-benefit analysis.^53^

In an attempt to reduce infections by the novel coronavirus, physical therapy professional organizations in Brazil have allowed their members to use telemedicine, teleconsultation, and telemonitoring to assist and support patients who require physical interventions.^54^ This allows professionals to limit their physical contact with patients to cases that require a physical examination or specific procedures.

### PHYSICAL THERAPY IN HOSPITALIZED PATIENTS WITH COVID-19

Studies show that 80% of patients diagnosed with COVID-19 do not require hospitalization. The remaining 20% of patients are hospitalized with severe (15%) or critical (5%) forms of the disease, and require access to ICUs.^55^ To date, the most common symptoms in patients hospitalized for COVID-19 include fever, cough, shortness of breath, muscle aches, mental confusion, headaches, sore throat, rhinorrhea, chest pain, diarrhea, nausea, and vomiting.^4,56^ An analysis of 1,099 people with COVID-19 admitted to hospitals in China found that lymphopenia and ground-glass opacity were present in 83.2 and 56.4% of patients, respectively.^21^

Patients with severe forms of the illness have impaired respiratory health, with symptoms such as dyspnea, persistent chest tightness, O_2_ saturation less than 95% in ambient air, PaO_2_/FiO_2_ less than 300 mm Hg, cyanosis, and high fever, which characterize SARS.^46^ In some cases, myalgia, rhinorrhea, headaches, and bilateral pneumonia have also been observed. Patients who experience respiratory complications must be admitted to intensive care and receive mechanical ventilation for respiratory support.^4,55^

Critical patients, on the other hand, display respiratory failure, septic shock, and multiple organ failure.^46,55^ Patients in critical condition also have different degrees of muscle dysfunction and cognitive impairment.^12,56^ A descriptive exploratory analysis of data from the Chinese infectious disease information system revealed a lethality of 49% in critical cases. Patients with comorbidities (cardiovascular disease, diabetes, chronic respiratory illness, hypertension, and cancer) had higher lethality rates (10.5, 7.3, 6.5, 6 and 5.6%, respectively) than those with no comorbidities.^24^

Therefore, patients with COVID-19, especially those with fragile health, must be rehabilitated by a multidisciplinary team.^18,57^ Physical therapists have been highly demanded in hospital settings during the novel coronavirus pandemic since these professionals make important contributions to treatment and recovery in both the early and late stages of illness for hospitalized patients with COVID-19.^15^

Patients admitted with moderate symptoms can also benefit from physical therapy as a means to prevent symptom aggravation; in these cases, physical therapists also perform a constant assessment of the need for respiratory physical therapy.^53^ In patients with a productive cough, which according to the literature, account for 34% of cases,^21^ respiratory training should be prescribed to increase airway permeability and prevent the accumulation of bronchial secretions.^25,53^ These techniques are thought to improve respiratory mechanics by increasing dynamic lung compliance.^58^ Such treatments are essential, especially for patients with preexisting comorbidities that can lead to hypersecretion or ineffective coughing, such as neuromuscular or respiratory disease, or cystic fibrosis.^25^

However, the role of the physical therapist is not restricted to the respiratory system: these professionals play a crucial role for hospitalized patients in the acute stages of illness, as they help minimize or neutralize the negative effects of immobility during hospitalization.^15^ In these cases, physical therapists perform exercises to increase peripheral muscle strength, change patient positions and promote physical movement to keep the patient active and minimize musculoskeletal complications. However, this type of activity should only be carried out if the patient’s clinical condition allows it;^50^ there is, as such, a need for continuous monitoring, especially of SpO_2_ values, to guarantee the safety of the intervention.^53^

In severe cases, physical therapy is a crucial part of intensive care. Physical therapists are part of the team responsible for the operation of ICUs since they play a crucial role in the management of patients with severe disease who require respiratory support. Their role extends from the early stages of preparation and adjustment of the ventilator, until intubation, weaning, and extubation.^59,60^ Physical therapists also develop procedures to prevent and/or treat common complications observed in ICU patients, such as neuropathy, myopathy, contractures, thrombosis, and postural instability.^10,11^

According to Wujtewicz et al.,^61^ patients with severe disease tend to develop pneumonia, and rapidly progress to acute hypoxemic respiratory failure and severe acute respiratory distress syndrome, requiring supplementary oxygenation. Previous studies have also reported on patients with spontaneous ventilation who suddenly went on to require intubation and mechanical ventilation. Therefore, most if not all patients with severe cases of COVID-19 require oxygen therapy or invasive mechanical ventilation (IMV), which in turn demands intensive physical therapy.^59^

IMV has been associated with several complications, such as baro/volutrauma, conditioned extinction of upper airway defense mechanisms, need for sedoanalgesia, and increased likelihood of infection. Furthermore, many patients do not tolerate noninvasive mechanical ventilation (NIMV) and develop issues such as skin lesions, eye irritation, dry mucosa, claustrophobia, and lung injury caused by excess oxygen.^62,63^ These data are concerning, as a study of 302 patients with MERS showed that most (92%) patients who received NIMV required intubation and IMV.^64^ A study performed by Arabi et al.^65^ also demonstrated that the time taken to initiate invasive ventilation may have contributed to the high number of deaths. Nevertheless, some studies have found that the high tolerability and comfort of NIMV is associated with the masking of more severe situations and the delayed provision of adequate ventilatory support.^62^ Since these treatments have both risks and contraindications, they should be performed by trained professionals; only a highly experienced team can manage the particularities of COVID-19 patients and minimize the risks of these procedures.^66^ Physical therapy interventions such as early mobilization must also be implemented to reduce the severity of the muscle atrophy incurred during intensive care, promote rapid functional recovery and improve independence for activities of daily living. According to several authors, the loss of mobility in patients treated in ICUs can harm several structures and systems, including the respiratory and cardiovascular systems, the muscles, as well as the skin and bones, with impairments beginning within 72 hours of admission.^67^ In the first 7 days of strict bed rest, muscle strength can decrease by up to 30%, with further decreases of 20% observed in each subsequent week. This restriction leads to alterations in muscle fibers, as well as peripheral and respiratory muscle atrophy, which can hinder extubation, prolonging the need for MV.^68^

For these reasons, the demand for physical therapists in hospital settings has steadily increased during the COVID-19 pandemic. The work of these professionals in the treatment and recovery of individuals with COVID-19 admitted to health care services, in both the early and late stages of the disease, promotes faster recovery and hospital discharge.^15^ It is crucial that these treatments be offered to patients, especially those who must return to their occupations as soon as possible. Furthermore, studies show that most patients infected with COVID-19 are actively employed: according to epidemiological studies, the symptoms of COVID-19 are less severe in children than in adults, and only a small share of individuals younger than 19 years develop severe (2.5%) or critical (0.2%) forms of the disease.^38^

### PHYSICAL THERAPY IN POST-ICU RECOVERY AND AFTER HOSPITAL DISCHARGE

Although little is known of the clinical consequences of COVID-19, experts have drawn attention to the long-term effects of ICU admission. ICU survivors with critical illnesses can develop what is known as “post-intensive care syndrome” or “post-ICU syndrome.”^11^ This condition is characterized by physical, cognitive, and psychological alterations that can reduce quality of life and interfere with the return to work.^69^

Myhren et al.^70^demonstrated that 55% of previously active ICU survivors who recover from severe illness return to work or school in the year after discharge. Kamdar et al.^71^ also demonstrated that, in a sample of 922 survivors of acute respiratory distress syndrome (ARDS) in 43 American hospitals, 44% of patients were unemployed after one year of hospital discharge. The study also noted a 71% reduction in patients’ financial earnings, while the variables most closely associated with unemployment were age and length of hospitalization. According to Simpson and Robinson,^11^ prolonged immobility is associated with cardiorespiratory deconditioning, postural instability, venous thromboembolism, muscle shortening, as well as myogenic, neurogenic and arthrogenic contractures.

It has also been established that interactions between comorbidities, preexisting chronic illnesses, and complications of acute illness, such as hypotension, hypoxia, hypo- or hyperglycemia, and polyneuromyopathy can contribute to the occurrence of symptoms associated with post-intensive care syndrome in ICU survivors.^72^ In some clinical groups, up to 100% of ICU survivors experience some degree of cognitive impairment which persists after hospital discharge.^73^ Surviving acute critical illness does not necessarily imply a return to baseline quality of life after hospital discharge.

These findings have resulted in growing concern regarding COVID-19 survivors who required prolonged hospitalization and ICU admission. Though there is still limited information on the nature and prevalence of post-ICU symptoms experienced by patients with COVID-19, some studies have identified deleterious consequences of prolonged hospitalization in these patients, including cognitive alterations, depression, anxiety, changes in mobility, and delirium, in addition to cardiovascular and pulmonary alterations.^26,74^

A 2-year retrospective observational study performed in the ICU of a hospital in Portugal demonstrated that most patients with ARDS go on to have severe clinical disease and require prolonged hospitalization. The same study found that 27.5% of cases of ARDS were associated with in-hospital complications, the most frequent of which were ventilation-associated pneumonia, pneumothorax, and ICU-acquired myopathy.^75^ Furthermore, studies of ARDS survivors, regardless of cause, have confirmed that these patients continue to have compromised health, functional impairments, reduced quality of life, and high health care costs even after ICU discharge.^69,76^

Evidence of long-term consequences in COVID-19 survivors continues to emerge in the literature. A prospective cohort study performed in Wuhan, China, examined the clinical outcomes of 131 patients (aged 18 to 88) who were discharged from hospital after having COVID-19 (severe and non-severe). The study found that 40.46% of patients had symptoms such as cough, fatigue, expectoration, tightness in the chest, dyspnea, dizziness, palpitations, and lymphopenia. Observational follow-up concluded that 48.09% of patients still had one or more of these symptoms in the first to second week post-discharge. This figure decreased to 13.74% by weeks 3 and 4, but many patients still presented with cough (9.16%), dyspnea (1.53%), pharyngeal pain (1.53%), and nausea (0.76%).^77^ Halpin et al.^27^ performed a study of 100 COVID-19 survivors discharged from a large teaching hospital in England (the Leeds Teaching Hospitals NHS Trust) and found that at 4 and 8 weeks post-discharge, patients continued to experience symptoms, the most common of which were muscle fatigue (72% in the ICU group and 60.3% in the ward group), shortness of breath (65.6% in the ICU group and 42.6% in the ward group) and psychological distress (46.9% in the ICU and 23.5% in the ward group).

This is a cause for concern since a follow-up study of SARS survivors revealed that lung damage and functional impairment showed the greatest recovery after 2 years of rehabilitation. The same study also observed using computed tomography that lung and femoral head damage could still be observed in some patients 15 years after hospital discharge.78 As a result, many authors believe that rehabilitation will constitute the second stage of recovery for COVID-19 survivors, with physical and occupational therapists playing a crucial role in the process.^15,74^

On a similar note, a study of COVID-19 survivors demonstrated that those who participated in a 6-week rehabilitation program showed improvements in lung function, functional capacity, and quality of life relative to a control group.^28^ Furthermore, a document titled “Rehabilitation consideration during the COVID-19 outbreak,” published by the Pan-American Health Organization, clarified the role of rehabilitation in the long-term management of patients with COVID-19.^26^

It is therefore crucial that rehabilitation professionals provide continued follow-up to patients infected with SARS-CoV-2, especially those who required intensive care and prolonged hospitalization, since, as the literature shows, COVID-19 can have short-, medium- and long-term effects on functioning, thereby interfering with the return-to-work process.

The present study revealed that COVID-19 affects a significant proportion of the active working population, and can have physical, psychological, and cognitive consequences that result in functional disability, especially for individuals who require prolonged hospitalization and intensive care. The literature has shown that patients with SARS, whether or not it is caused by the novel coronavirus, require physical therapy to prevent and recover from issues that can persist in the short, medium, and long term. Physical therapists must be involved in the battle against this illness to help patients recover their physical function and return to work as quickly, safely, and effectively as possible. Furthermore, though a large number of studies have examined occupational health in association with COVID-19, few have described patients’ occupational activities, which may result in the underreporting of cases and an ineffective analysis of the impact of the illness on different occupational classes.
